# Clustering-Based Accelerometer Measures of Physical Activity Patterns in Children With Overweight or Obesity: Baseline Cross-Sectional Methodological Analysis

**DOI:** 10.2196/87532

**Published:** 2026-10-08

**Authors:** Hyatt Moore IV, Thomas N Robinson, Alexandria Jensen, Fatma Gunturkun, K Farish Haydel, Kristopher I Kapphahn, Manisha Desai

**Affiliations:** 1 Naval Postgraduate School, 1 University Circle, Monterey, CA, 93943, United States; 2 Quantitative Science Unit, Stanford University, Stanford, CA, United States; 3 Department of Pediatrics, Stanford Solutions Science Lab and Division of General Pediatrics, Stanford University, Palo Alto, CA, United States; 4 Department of Medicine, Stanford Prevention Research Center, Stanford University, Palo Alto, CA, United States

**Keywords:** accelerometry, wearable sensors, physical activity, unsupervised machine learning, cluster analysis, obesity, pediatric health, waist circumference

## Abstract

**Background:**

Accelerometers produce high-resolution physical activity data, but commonly used summary measures often reduce these data to total volume, intensity, or variability and may not retain interpretable temporal structure across the day. Cluster-based summaries may provide a way to characterize daily physical activity profiles while preserving information about when activity occurs.

**Objective:**

This study evaluated whether cluster-derived accelerometer summary measures could represent daily physical activity patterns and explain variation in pediatric cardiometabolic outcomes comparably to traditional accelerometer summary metrics.

**Methods:**

This baseline cross-sectional methodological analysis used data from 268 Latino children with overweight or obesity from low-income families participating in the Stanford GOALS trial. Participants were aged 7 to 11 years old and wore accelerometers for a minimum of 1 week according to study protocol. Valid daily activity profiles were summarized within a 7:00 AM-11:00 PM analytic window using consecutive, nonoverlapping 10-minute intervals. Daily profiles were clustered using unsupervised learning, and participant-level cluster-derived measures were created from the distribution of valid days assigned to each cluster. We compared these measures with traditional accelerometer summaries, including time spent in activity intensity states, Time Active Mean, Time Active Variability, Activity Intensity Mean, and Activity Intensity Variability. Linear regression models were used to evaluate associations with waist circumference, fasting insulin, and fasting triglycerides, adjusting for age and sex. Model performance was compared using *R*^2^ and the Akaike information criterion.

**Results:**

Cluster-derived measures explained a comparable proportion of variation in the 3 cardiometabolic outcomes to traditional accelerometer summary metrics. For example, the highest *R*² values among the cluster-derived measures were 25%, 11%, and 6% for waist circumference, fasting insulin, and fasting triglycerides, respectively, compared with 25%, 10%, and 6% for Time Active Mean. No single summary-measure approach consistently yielded the highest *R*² across all 3 outcomes.

**Conclusions:**

Cluster-derived accelerometer measures provide a regression-ready approach for summarizing daily physical activity patterns while preserving interpretable clock-time structure. In this baseline analysis, these measures performed comparably to traditional accelerometer summaries while capturing temporal features not represented by conventional volume- or intensity-based metrics. Future work should evaluate external validation across diverse populations and settings and assess the use of these measures in longitudinal and intervention analyses.

## Introduction

### Measures of Physical Activity

Accelerometers have been increasingly used to measure physical activity objectively [1]. The richness of accelerometer data presents both challenges and opportunities. Modern accelerometers measure acceleration in 3 dimensions at a high frequency (eg, 40 Hz or more) and, often, for long periods of time (eg, 10 d). How to process and summarize high-resolution accelerometer data is an open topic. Accelerometer-based measures of physical activity may convert the accelerometer data to describe the time spent in activity states such as “percent time spent in a sedentary state.” Others summarize the data as mean *counts* per minute (CPM), where count—a unitless measure developed by ActiGraph [2]—reflects the level of intensity of activity at a given epoch or window of time (eg, a typical epoch may cover a 15-s interval) [3-11]. Several studies have demonstrated CPM to correlate with mechanical loading and activity [8-11]. Any measure that summarizes activity based on the raw accelerometer data requires some processing. For example, the count is obtained by filtering and then summing the raw acceleration over consecutive, nonoverlapping epochs (ActiGraph LLC). Other common approaches, termed *cut-point* methods, classify activity intensity using predetermined thresholds based on accelerometer signals [8,12-16]. These approaches have been systematically reviewed in youth by Kim et al [16]. Processing involves defining epochs, transforming raw data, and specifying cut-points to classify intensity. Figure 1 illustrates raw tri-axial accelerations, in units of gravity, over a 24-hour period from a child participating in the Stanford GOALS trial [17,18] along with its corresponding count values, CPM, and activity categories using Romanzini cut-point approach [8].

The initially proprietary nature of “count” data previously garnered some criticism by those who viewed the lack of standardization and transparency as a limitation. In their comprehensive investigation, Bai et al [19] addressed such limitations of many existing approaches that rely on counts. They proposed accelerometer-based summary measures derived from raw tri-axial acceleration signals rather than proprietary counts. These measures summarize 2 conceptual dimensions of activity: duration and intensity. Time Active Mean (TAM) and Time Active Variability (TAV) describe the mean and variability of time spent active, whereas Activity Intensity Mean (AIM) and Activity Intensity Variability (AIV) describe the mean and variability of activity intensity during active periods. These 4 measures provide a more transparent approach to the analysis of raw accelerometry data, although they still require a cut-point to distinguish activity from rest for the sample and accelerometer equipment under investigation, as described in the *Methods* section.

While these more recent methods offer greater transparency, there is still an opportunity to leverage the richness of the data to address certain types of research questions in new and innovative ways. For example, our team has been interested in questions that include the timing and patterns of physical activity that traditionally used measures may not reflect, such as whether individuals with the same amount of cumulative daily exercise who work out in the evening have more favorable cardiovascular health than those who work out in the morning. The summary measures described above do not address the pattern of physical activity signals across the day. In this work, we formally propose an alternative summary measure that leverages daily physical activity patterns preserving key temporal aspects of the accelerometer data. We evaluate the performance of the measure relative to traditional measures in a regression framework by examining the proportion of variation explained in 3 prespecified clinical outcomes.

**Figure 1.**
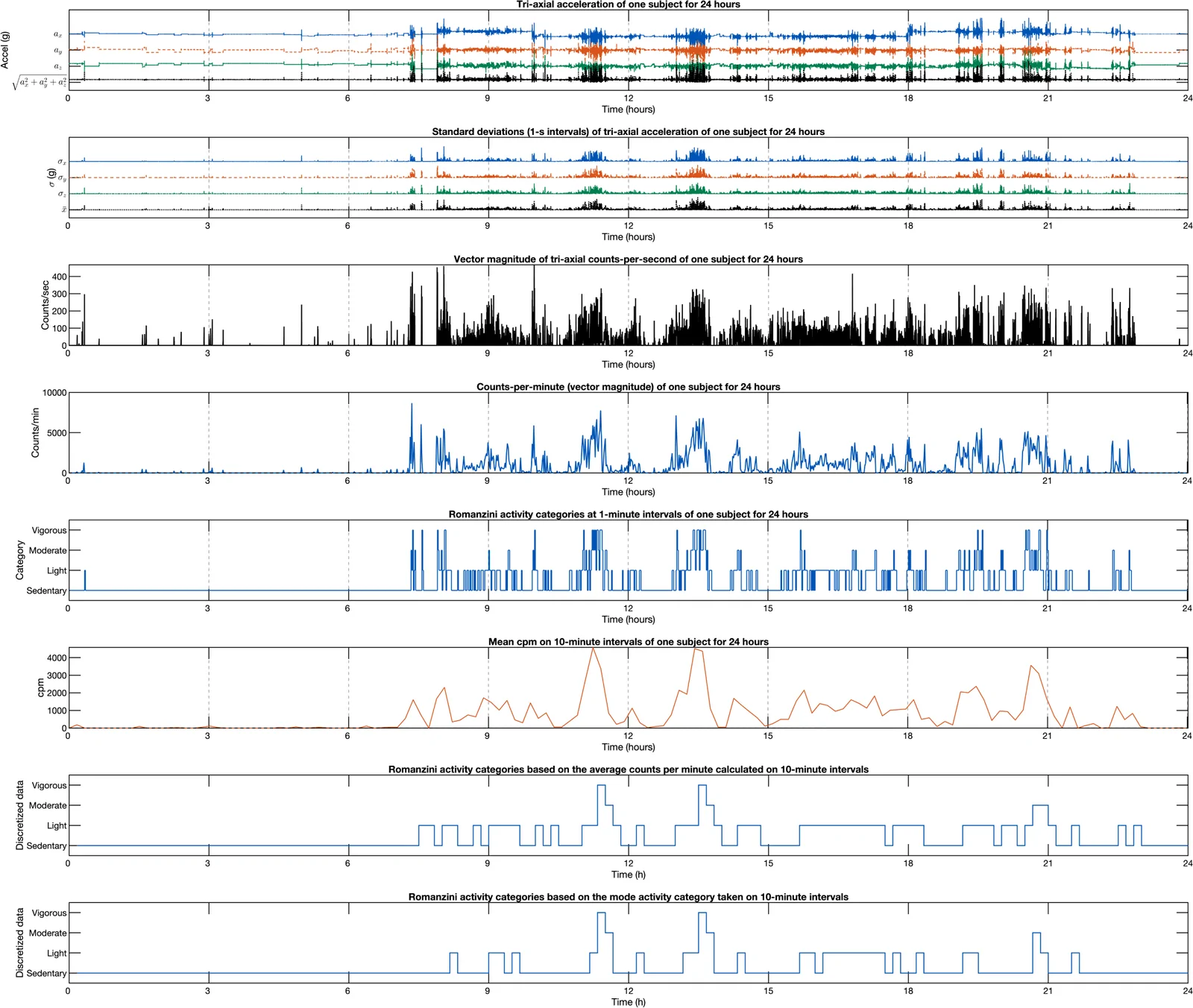
Overview of accelerometer-based activity visualization of a Stanford GOALS trial participant for a 24-hour period: (1) tri-axial acceleration signals and their vector magnitude (VM), (2) SDs of acceleration signals and their mean, (3) VM of count signal at 1-second intervals, (4) VM of count signal at 1-minute intervals (*counts* per minute [CPM]), (5) Romanzini activity categories at 1-minute intervals, (6) mean CPM of VM count signal in 10-minute frames, (7) Romanzini activity categories at 10-minute intervals based on mean CPM, and (8) Romanzini activity categories at 10-minute intervals based on the mode of 1-minute intervals.

### Clustering-Based Physical Activity Patterns

We are not the first to apply unsupervised machine learning to accelerometer data to gain insights into physical activity patterns. In a systematic review by Jones et al [20], 13 papers were identified that applied unsupervised machine learning to accelerometer data, and researchers have continued in this direction. For example, Nawrin et al [21] describe the need for measures that capture the temporal nature of intensively measured physical activity and demonstrate the diversity of physical activity patterns observed in a small dataset of 42 healthy individuals. Several studies have further associated patterns—discovered by machine learning techniques—with health outcomes such as obesity, cardiovascular disease (CVD), and mental health conditions within a regression framework [22-27]. These clustering-based methods provide a more granular perspective of physical activity patterns by capturing variations in the timing and intensity of activity throughout the day, rather than solely summarizing total physical activity volume. For instance, Niemelä et al [24] identified 4 temporal physical activity clusters in midlife adults and found that these patterns were significantly related to cardiovascular disease risk. Similarly, Nawrin et al [23] and Smagula et al [27] highlighted the role of physical activity timing in metabolic health and depression risk, reinforcing the idea that physical activity patterns—not just duration or intensity—may influence key health outcomes. These studies have demonstrated the promise of unsupervised machine learning approaches for gaining insight into physical activity patterns and their role in health.

There are methodological challenges, however, in using a summary measure derived from unsupervised machine learning within a regression context. As Jones et al [20] noted, the lack of consensus on analytic approach and feature selection limits comparability across studies and underscores the need for more transparent and standardized methods. Our study addresses this issue by formally evaluating cluster membership, derived from aligned daily activity profiles, as a physical activity summary measure in comparison with established summary measures from Romanzini et al [8] and Bai et al [19]. We further examine the sensitivity of cluster-based findings to data-processing decisions and assess performance within a regression framework focused on the proportion of variance explained in prespecified clinical outcomes. In this way, the study is designed to clarify when pattern-based measures may add value, particularly for research questions in which the timing of activity across the day is of substantive interest.

The aim of this study was to develop and evaluate clustering-based accelerometer measures derived from aligned daily activity profiles and to examine their relationships with key outcomes at baseline within the Stanford GOALS trial [17,18]. The methodological focus was to preserve interpretable clock-time structure across the day rather than reduce physical activity to aggregate volume measures alone. Accordingly, this paper is intended as a baseline cross-sectional methodological analysis, not as a longitudinal or intervention-effects study.

## Methods

### Study Design

This study was a secondary baseline cross-sectional methodological analysis using data from the Stanford GOALS trial, a community-based randomized controlled trial. The present analysis focused on deriving clustering-based accelerometer measures from daily activity profiles and evaluating their relationships with key outcomes at baseline. It was designed to characterize daily activity structure at a single study time point rather than to assess longitudinal change or intervention response.

### Stanford GOALS

The Stanford GOALS trial was a National Institutes of Health–funded, 3-year, community-based randomized controlled trial comparing strategies for weight control among 268 children aged 7 to 11 years with overweight or obesity from low-income, Latino (98%) families. Children had to be at or above the 85th percentile of the Centers for Disease Control and Prevention’s growth charts for their age and sex to participate [18]. The baseline sample used in the current analysis consisted of 121 male participants and 147 female participants with an average age of 9.53 (SD 1.46) years and BMI of 25.01 kg/m^2^ (Table 1).

Participants were provided with ActiGraph GTX3+ ambulatory monitors and instructed to wear them on their hip, continually, for a period of at least 7 days, except when bathing or swimming. The devices measured acceleration (gravity) on 3 perpendicular axes at a frequency of 40 Hz. Upon the completion of the baseline study period, the devices were returned, and the data were exported to comma-separated value (.csv) files using ActiGraph’s companion software, ActiLife, which derives additional *count* measures from the raw data. These include counts for each axis of acceleration (*x*, *y*, and *z*) as well as their vector magnitude (VM, defined as $\sqrt{x^{2}+y^{2}+z^{2}}$).

**Table 1.** Measures from Stanford GOALS trial participants taken at baseline^a^.

Measure	Male (n=121), mean (SD)	Female (n=147), mean (SD)	All (n=268), mean (SD)
Age (y)	9.49 (1.50)	9.57 (1.43)	9.53 (1.46)
Height (cm)	139.36 (9.83)	139.08 (9.67)	139.20 (9.73)
Weight (kg)	49.64 (12.53)	48.81 (12.99)	49.18 (12.77)
Waist (cm)	85.31 (10.79)	85.21 (11.03)	85.25 (10.90)
BMI (kg/m^2^)	25.20 (3.79)	24.85 (4.08)	25.01 (3.95)
Insulin (µIU/mL)	13.79 (8.86)	17.45 (12.54)	15.80 (11.16)
Triglycerides (mg/dL)	90.00 (53.97)	105.1 (56.46)	98.28 (55.75)

a Waist circumference, fasting insulin levels, and fasting triglyceride levels were selected for our analysis measures.

### Ethical Considerations

The parent Stanford GOALS trial was approved by the Stanford University Administrative Panel on Human Subjects in Medical Research (protocol #19311). Parent or guardian written informed consent and Health Insurance Portability and Accountability Act authorization were obtained, and child assent was obtained as part of the parent trial. The parent trial was registered at ClinicalTrials.gov (NCT01642836). The present manuscript reports a baseline cross-sectional secondary methodological analysis of deidentified accelerometer and clinical data collected during the Stanford GOALS trial and does not evaluate longitudinal or intervention effects.

### Physical Activity Data

This baseline analysis used accelerometer data collected from 268 boys and girls participating in the Stanford GOALS trial. Participants were instructed to wear ActiGraph GT3X+ accelerometers on the hip continuously for at least 7 days, except when bathing or swimming. Count data for the *x-*, *y-*, and *z*-axes and their vector magnitude were exported in consecutive, nonoverlapping 1-minute epochs. Raw acceleration signals were summarized by calculating the SDs of each axis in consecutive, nonoverlapping 1-sec intervals. Before screening for nonwear or device malfunction, the accelerometer files yielded 2322 candidate 24-hour participant-days for processing.

### Data Quality

Daily accelerometer profiles were constructed within a prespecified analytic window of 07:00 to 23:00. This window was selected as part of the accelerometer-processing workflow to support aligned daily-profile analyses while retaining adequate participant-level profile availability; candidate windows and resulting profile-retention summaries are presented in Table S1 (Multimedia Appendix 1). Nonwear was identified from the count data at 1-minute resolution using Choi method [28]. Periods of device malfunction were identified in the raw gravity signal along the *x*-, *y*-, and *z*-axes using custom code by clustering the raw signal and examining outlying groups for shared signal patterns, which were subsequently confirmed by ActiGraph’s support team as device malfunction. Profiles were flagged and excluded from the analyses if nonwear or device malfunction was detected within the selected analytic window. Within the 7:00 AM to 11:00 PM window, 1009 of 2322 (43.5%) candidate daily profiles were flagged during screening, leaving 1313 (56.5%) valid daily profiles; 256 (95.5%) participants retained at least 1 valid daily profile, whereas 12 (4.5%) participants retained none. Baseline clinical outcome data were complete for all 268 participants; missingness in the present analysis was therefore limited to accelerometer-derived daily profiles affected by nonwear or device malfunction within the analytic window.

### Accelerometer Summary Measurements

#### Physical Activity Categories: Cut-Point–Based Summary Measures

CPMs were calculated from the vector-magnitude of the GT3X tri-axial count data and categorized as sedentary behavior (SB), light physical activity (LPA), and moderate-to-vigorous physical activity (MVPA), a combination of moderate physical activity (MPA) and vigorous physical activity (VPA). The cut-points for these categories were taken from Romanzini et al [8]. Every minute from 7:00 AM to 11:00 PM was categorized in this way, and the total amount of time spent in each category was tabulated per subject-day. The average amount of time spent in each physical category was derived from the tabulated activity data of each subject.

#### Daily Accelerometer Summary Measures

Accelerometer summary measures—TAM, TAV, AIM, and AIV—are calculated from variation measured from the accelerometers’ raw signal data as outlined in Bai et al [19] for each day. Achieving this requires the use of a binary activity label, *L(t*), which categorizes accelerometer readings at time *t* as either active (1) or inactive (0). This label is obtained on a 1-second interval by comparing the SD of the accelerometer signal to a cut-point, C, which is determined from the data. Bai and others did this in their data by examining density and cumulative distributions of the SD and then selecting the point at which there was a clear, visible flattening of the density. We followed the same methodology and selected C=0.6 based on our results (Figure S1 in Multimedia Appendix 1). TAM and TAV are, respectively, the mean and SD of the time spent active (*L(t)=1*) for each person for each day examined. This gets at the duration of time spent active, but not the intensity of the activity. Activity intensity is defined as the SD of the original signal relative to the average standard deviation of the signal when inactive, which is calculated per subject-day. The AIM and AIV metrics are the average and SDs, respectively, of the activity intensity signal during active periods. Tables S2 and S3 (Multimedia Appendix 1) present these metrics for 3 Stanford GOALS participants to help orient readers to the dataset.

#### Daily Physical Activity Patterns

In this study, we propose a metric based on patterns of activity. We used *k*-means clustering to categorize each participant’s daily activity profile within a 7:00 AM to 11:00 PM analytic window using average counts from consecutive, nonoverlapping 10-minute intervals. This window was selected from candidate windows as part of the prespecified processing workflow to retain broad daytime and evening coverage while maintaining participant-level profile availability (Table S1 in Multimedia Appendix 1). The 16-hour duration also allowed the sorting variants to partition the analytic day evenly into 1-, 2-, 4-, 8-, and 16-hour segments. Representing each valid day in 10-minute intervals yielded a 96-element profile vector for each day, with the number of retained profiles varying across participants. This interval length was used to preserve interpretable clock-time structure while reducing sensitivity to minute-level irregularity.

Figure 2 illustrates how these profiles can be clustered to identify common patterns of daily activity within the group. In the unsorted profiles shown in Figure 2, cluster 1 was characterized by comparatively low activity throughout the day, Cluster 2 by higher activity from the morning through late afternoon, and Cluster 3 by a pronounced late-afternoon and evening increase, peaking near 7:00 PM. Although overall activity magnitude contributed to cluster differentiation, timing also contributed: the Cluster 2 and Cluster 3 centroids crossed in the late afternoon and exhibited their highest activity during different portions of the day. We therefore interpreted the clusters as recurring activity-profile patterns rather than as definitive latent classes. The sorting variants (Sort_01, Sort_02, Sort_04, Sort_08, and Sort_16) were evaluated as a structured sensitivity analysis, spanning broader to more localized within-day sorting, to examine how partial relaxation of exact clock-time ordering affected clustering results while preserving differing degrees of temporal structure.

Clustering was performed using Padaco, a publicly available MATLAB-based accelerometer visualization and analysis program with clustering functionality. Daily profile vectors were clustered using k-means with squared Euclidean distance. This approach compares activity at corresponding positions in the represented daily profiles and therefore preserves the intended clock-time interpretation. An elastic alignment measure such as dynamic time warping was not used because the scientific objective was not to treat profiles as equivalent after temporal warping across the day. Instead, limited tolerance for differences in the precise timing of activity was introduced explicitly through the within-segment sorting variants described below. To support the reproducibility of the clustering solution, we used Padaco’s reproducibility setting, which initializes the MATLAB random number generator with rng('default') before clustering. The Padaco source code is available at GitHub [29].

To estimate the number of clusters, *k*, we used the prediction strength metric developed by Tibshirani and Walther [30], together with the recommendations of Yu et al [31]. We calculated the prediction strength statistic for up to *k*=50 and selected the highest value of *k* above 0.75 or, when applicable, a local maximum or elbow in the prediction-strength curve. In this study, we used counts for comparison, but the same approach can be applied to other time-series units, including raw acceleration, monitor independent movement summaries, and related measures. To visually assess the clustering solution, we projected the 96-bin daily activity profiles into 2 dimensions using principal component analysis (PCA) and overlaid the assigned cluster labels. This PCA projection was used only as a post hoc visual diagnostic of cluster structure and was not used in the clustering algorithm or subsequent regression analyses.

**Figure 2.**
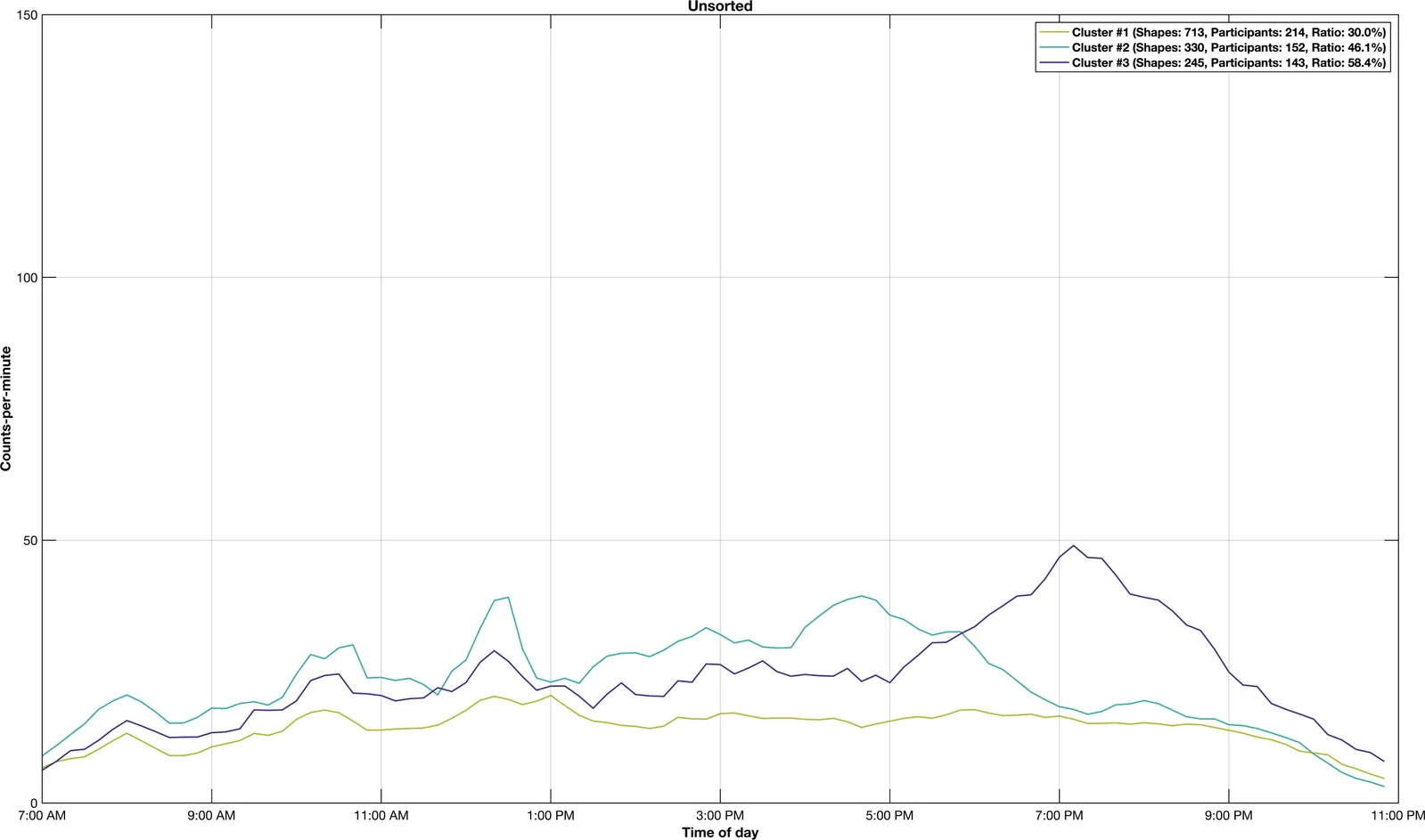
Cluster-derived daily physical activity profiles from accelerometer count data summarized in consecutive, nonoverlapping 10-minute intervals from 7:00 AM to 11:00 PM. The profiles illustrate 3 recurring patterns identified from the unsorted daily activity data: low activity across the day, higher activity from morning through late afternoon, and relatively higher evening activity. The legend reports the number of daily profiles assigned to each cluster, the number of unique participants contributing profiles to the cluster, and the participant-to-profile ratio.

#### Sorting Activity Levels Prior to Clustering

Minor shifts in the precise timing of activity could cause otherwise similar days to appear dissimilar when profiles are compared at corresponding 10-minute intervals. At the same time, fully aligning temporal features across the day could remove substantively meaningful distinctions between activity occurring in different portions of the day. We therefore sorted activity values by magnitude within prespecified consecutive time segments while retaining the order of those segments before applying k-means clustering. This preprocessing allowed profiles with minor within-segment timing differences—for example, a brief morning activity peak at 8:00 AM versus 8:30 AM—to remain similar, while preserving distinctions between activity occurring in broader portions of the day. For example, it continued to distinguish a profile characterized by high morning activity and low afternoon and evening activity from one characterized by high afternoon activity and lower morning and evening activity. A limiting version of this approach is to sort the vectors from the highest to the lowest activity values *over the whole day*, which removes clock-time ordering while retaining information about the level and distribution of activity across the analytic window. We included this whole-day sorting approach (Sort_01) as an extreme comparison condition in the analysis. To preserve progressively more temporal structure, we also sorted activity values within prespecified time segments: two 8-hour segments (Sort_02), four 4-hour segments (Sort_04), eight 2-hour segments (Sort_08), and sixteen 1-hour segments (Sort_16; Figure 3). Figure 3 shows the cluster centroids for the unsorted and sorted representations used in the analysis, illustrating the recurring activity-profile categories from which participant-level membership variables were derived.

**Figure 3.**
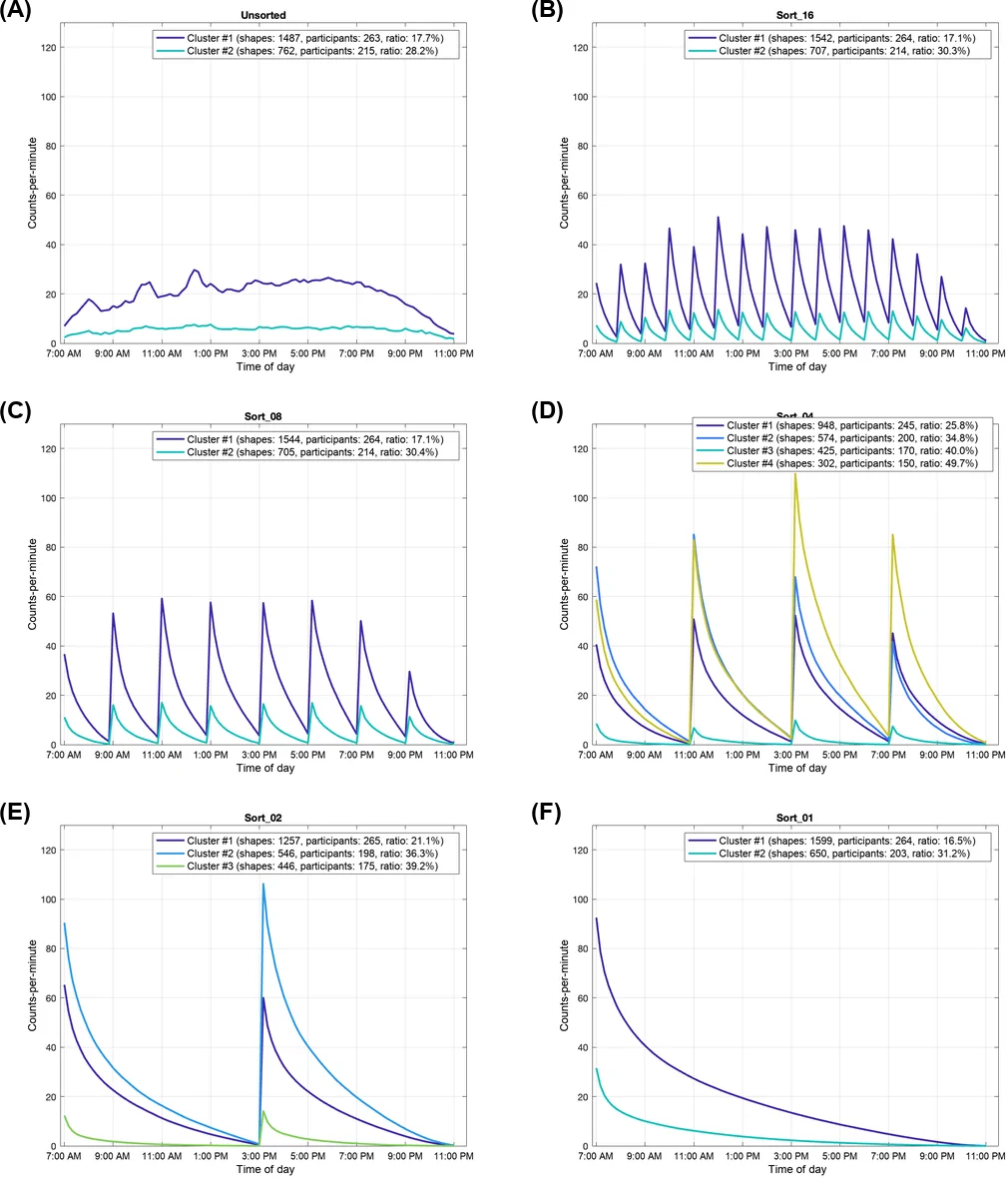
Daily activity profiles found by sorting accelerometer data, within consecutive segments of the day, prior to clustering. Accelerometer count data on 10-minute intervals from 7:00 AM to 11:00 PM were sorted for each participant prior to clustering. (A) Unsorted. (B) Sort_16: daily accelerometer data are first split into 16 consecutive 1-hour segments that are individually sorted prior to clustering. (C) Sort_08: daily accelerometer data are first split into 8 consecutive 2-hour segments that are each sorted. (D) Sort_04: daily accelerometer data are split into 4 consecutive 4-hour segments, which are locally sorted prior to clustering. (E) Sort_02: daily accelerometer data are split into 2 consecutive 8-hour segments, which are each sorted prior to clustering. (F) Sort_01: accelerometer data are sorted across the entire day prior to clustering. Temporal information is discarded in this scenario.

### Regression Models and Metrics for Comparing Summary Measures

#### Model Comparison Framework

To evaluate the proposed cluster-derived summary measure and its sorting-based variations, we compared the variation in a given outcome explained by each of the candidate accelerometer summary measures using the coefficient of determination, *R*^2^. We selected 3 clinical outcomes for the primary regression analysis: waist circumference, fasting triglyceride levels, and fasting insulin levels. These outcomes were selected because they represent clinically relevant markers of central adiposity, lipid metabolism, and insulin-related metabolic risk in pediatric obesity, consistent with pediatric cardiovascular risk frameworks [32]. Each outcome was modeled as the dependent variable in a linear regression model that included the accelerometer-derived physical activity measure of interest and adjusted for age and sex. Model performance was summarized using *R*^2^ and Akaike information criterion (AIC). *R*^2^ was used to describe the proportion of variation explained, whereas AIC was used to compare relative model fit among candidate models with the same outcome.

#### Modeling Outcome as a Function of Cut-Point–Based Summary Measures

To compare the proportion of outcome variation explained by traditional accelerometer summary measures, each clinical outcome was modeled as a function of the participant-level physical activity measure while adjusting for age and sex. The physical activity measures included the duration spent in each cut-point–based physical activity category defined by Romanzini et al [8] and the daily accelerometer summary measures proposed by Bai et al [19]. Each candidate measure was summarized across the valid observed days contributed by the participant and evaluated in a separate regression model. For example, 1 model regressed the clinical outcome on the participant’s average sedentary behavior across valid observed days, while adjusting for age and sex:


$$Y_{i}=\beta_{0}+\beta_{1}\cdot A_{i}+\beta_{2}\cdot S_{i}+\beta_{3}\cdot {AverageSB}_{i}+\varepsilon_{i}\;$$

where *Y_i_* is the clinical outcome for participant *i*, *A_i_* is age, *S_i_* is sex, AverageSB*_i_* is the participant-level average sedentary behavior measure, and *ε*_*i*_ is the error term.

#### Modeling Outcome as a Function of Cluster Membership Distribution

A linear regression framework was also used to model each clinical outcome as a function of cluster membership distribution while adjusting for age and sex. For each clustering solution, participant-level cluster membership was represented as the proportion of days assigned to each cluster. Because these proportions sum to 1 across clusters for each participant, 1 cluster-membership proportion was omitted as the reference category in each regression model. Specifically, the outcome was modeled as:


$$Y_{i}=\beta_{0}+\beta_{1}\cdot A_{i}+\beta_{2}\cdot S_{i}+\sum_{j=1}^{k-1}\beta_{j+2}\cdot C_{j,i}\;+\varepsilon_{i}\;$$

where *C_j,i_* is the proportion of days that participant *i* had in cluster *j* relative to the total number of valid days observed for participant *i*, such that $\sum_{j=1}^{k}C_{j,i}=1$. For example, if the total clusters are *k*=3 and a given participant had 7 days of activity, with 4 days belonging to cluster #1, 3 days belonging to cluster #2, and zero days belonging to cluster #3, then their cluster membership distribution would be C_1_=4/7=0.5714, C_2_=3/7=0.4286, and C_3_=0. Thus, we are not constrained by the total number of clusters or differences in the total number of days available for each participant, which may vary (eg, removal due to data cleaning), because they are normalized according to the participant’s total contributions (7 in the example given). This modeling approach was applied to the clusters defined by each of our preprocessing approaches: unsorted and sorted, in day-wise partitions of 1, 2, 4, 8, and 16 hours.

## Results

The analytic sample for this baseline cross-sectional analysis was drawn from participants in the Stanford GOALS trial with accelerometer data meeting the prespecified daily-profile screening criteria. Under the selected 7:00 AM to 11:00 PM analytic window, 1313 valid daily profiles remained after screening, and 256 participants contributed at least 1 valid daily profile for the clustering-based analyses. Table 2 presents variance explained (*R*^2^) and goodness of fit (AIC) corresponding to each candidate summary measure when modeling waist circumference, fasting insulin levels, and fasting triglyceride levels; values for a model using only age and sex are included for reference as well.

Across outcomes, the results were broadly comparable across candidate summary measures. No single summary-measure approach consistently yielded the highest *R*² across all 3 outcomes. For example, the proportion of variation in insulin explained ranged from only 0.0636 (LPA) to 0.1064 (VPA) for the physical activity categories. The daily accelerometer summary measures have a similar level of variation explained and range from 0.0618 (AIV) to 0.1024 (TAM). Cluster member distribution explains from 0.0872 for the unsorted clusters to 0.1051 for Sort_02. Further, the ability of all measures to explain variation varied widely depending on the clinical outcome. For example, the unsorted cluster membership distribution explains only 0.0221 of the variation observed in triglycerides, 0.0872 of insulin variation, and a proportion of 0.2300 in waist circumference variation. Across all summary measures, the proportion of variation explained was the highest for waist circumference, followed by fasting insulin and then fasting triglyceride levels.

We additionally evaluated how alternative preprocessing or sorting methods prior to clustering influenced model performance for each outcome. When modeling with cluster membership distribution, variation explained was comparable across sorting approaches. However, sorting the days in eight 2-hour sections (Sort_08) prior to clustering yielded the best results for waist circumference (*R*^2^=0.2516, AIC=1880.5), while presorting in two 8-hour sections (Sort_02) had the highest *R*^2^ for insulin (*R*^2^=0.1051, AIC=1949.4) and presorting within four 4-hour sections was best for triglycerides (*R*^2^=0.0585, AIC=2763.6).

**Table 2.** Age- and sex-adjusted regression models comparing physical activity summary measures as predictors of waist circumference, fasting insulin, and fasting triglycerides in Stanford GOALS participants^a^.

Health outcome and statistic	Age + sex covariate	Physical activity categories					Accelerometer summary measures				Cluster membership					
		SB^b^	LPA^c^	MVPA^d^	MPA^e^	VPA^f^	TAM^g^	TAV^h^	AIM^i^	AIV^j^	Unsorted	Sort_01	Sort_02	Sort_04	Sort_08	Sort_16
Waist (cm)																
*R*^2^	0.1911	0.2422	0.2332	0.2183	0.2039	0.2238	0.2539	0.2418	0.2349	0.2149	0.2300	0.2348	0.2281	0.2190	0.2516	0.2266
AIC^k^	1869.2	1881.7	1884.7	1889.6	1894.3	1887.8	1850.8	1854.9	1857.2	1863.7	1887.8	1886.1	1890.4	1895.4	1880.5	1888.9
Insulin																
*R*^2^	0.0522	0.0786	0.0636	0.0953	0.0735	0.1064	0.1024	0.0956	0.0796	0.0618	0.0872	0.0970	0.1051	0.1011	0.0995	0.0937
AIC	1925.5	1952.8	1957.0	1948.1	1954.2	1945.0	1913.8	1915.7	1920.1	1925.0	1952.4	1949.7	1949.4	1952.5	1949.0	1950.6
Triglyceride																
*R*^2^	0.0143	0.0238	0.0223	0.0256	0.0225	0.0288	0.0555	0.0484	0.0449	0.0300	0.0221	0.0227	0.0344	0.0585	0.0230	0.0221
AIC	2742.1	2766.9	2767.3	2766.4	2767.2	2765.5	2733.5	2735.2	2736.2	2740.1	2769.3	2769.2	2768.1	2763.6	2769.1	2769.3

a Measures include cut-point–based activity categories, daily accelerometer summary measures, and cluster-derived measures based on 10-minute count profiles from 7:00 AM to 11:00 PM. Sorting approaches are described in the *Methods* section. Activity categories include sedentary behavior, light physical activity, moderate-to-vigorous physical activity, moderate physical activity, and vigorous physical activity. Daily accelerometer summary measures include Time Active Mean, Time Active Variability, Activity Intensity Mean, and Activity Intensity Variability.

b SB: sedentary behavior.

c LPA: light physical activity.

d MVPA: moderate-to-vigorous physical activity.

e MPA: moderate physical activity.

f VPA: vigorous physical activity.

g TAM: Time Active Mean.

h TAV: Time Active Variability.

i AIM: Activity Intensity Mean.

j AIV: Activity Intensity Variability.

k AIC: Akaike information criterion.

PCA overlays showed that the assigned clusters generally occupied different regions along continuous distributions of daily activity profiles across the preprocessing specifications considered (Supplementary Figure S2 in Multimedia Appendix 1). The distribution of variance across PC1 and PC2 changed with the preprocessing step, indicating that the sorting specification affected which aspects of the transformed daily activity profiles were emphasized in the reduced 2D space. Sort_02 showed stronger organization along PC1, whereas Sort_04 showed additional organization along PC2. These projections were used as visual diagnostics of the cluster assignments and illustrate their organization in 2 dimensions but were not used to define the clustering solutions or interpreted as evidence of discrete latent groups.

When modeling with physical activity categories, the duration of SB was best for waist circumference (*R*^2^=0.2422, AIC=1881.7), while the duration of VPA was best for insulin (*R*^2^=0.1064, AIC=1945.0) and triglycerides (*R*^2^=0.0288, AIC=2765.5). When using Bai’s physical activity summary measures, TAM performed best for modeling each outcome: waist circumference (*R*^2^=0.2539, AIC =1850.8), insulin (*R*^2^=0.1024 and AIC=1913.8), and triglycerides (*R*^2^=0.0555, AIC=2733.5).

## Discussion

This baseline cross-sectional methodological analysis evaluated a clustering-based approach for summarizing accelerometer-derived daily physical activity profiles in the Stanford GOALS trial. The primary contribution is the development and evaluation of cluster-derived participant-level measures that preserve interpretable clock-time structure across the day while remaining usable in conventional regression models. Across the cardiometabolic outcomes considered, these cluster-based measures explained a comparable proportion of variation to traditional accelerometer summary metrics. Although several physical activity summary measures showed statistically detectable associations with cardiometabolic outcomes, the absolute variance explained by these models was modest, especially for triglycerides. This finding is expected, as triglyceride levels and related cardiometabolic markers are influenced by many factors beyond accelerometer-derived activity patterns, including diet, adiposity, genetics, pubertal status, medication use, sleep, and other behavioral or clinical characteristics. Therefore, small differences in *R*^2^ or AIC across models should not be overinterpreted. These comparisons are most useful for showing that cluster-derived measures can be incorporated into conventional regression models and can perform comparably to traditional accelerometer summaries while preserving interpretable temporal structure, rather than for establishing the stand-alone clinical predictive utility of any single measure.

Summarizing accelerometer-derived physical activity patterns in a meaningful way remains a challenge in the field. Traditional summary measures, including cut-point–based classifications and intensity-derived metrics, capture important dimensions of total activity volume, intensity, and variability, but they generally do not retain the sequence and timing of movement across the day [19]. Recent studies using clustering and related temporal-pattern approaches have shown that the timing, regularity, and daily distribution of physical activity may provide information beyond total activity volume alone [21,22,24,27]. Other work has also linked later or more irregular activity patterns with poorer behavioral or metabolic profiles [23,27]. The present study builds on this growing literature by evaluating whether clock-time daily activity patterns can be converted into regression-ready participant-level measures and compared directly with traditional accelerometer summaries.

The methodological motivation for this approach also draws from profile-clustering applications in other time-series domains. In civil engineering and energy systems, load-shape clustering has been used to summarize recurring temporal profiles from smart meter data and to support questions about energy-use patterns and optimization [33]. Here, we adapted that profile-based logic to accelerometer-derived daily activity patterns. Each valid day was represented as an aligned activity profile, clustered into common daily patterns, and then summarized at the participant level as the distribution of days assigned to each cluster. This construction is important because it allows temporal pattern information to be retained while producing predictors that can be incorporated into familiar regression frameworks.

The comparison with traditional accelerometer summaries suggests that cluster-derived measures can complement, rather than replace, existing metrics. Traditional measures are well suited for questions about total activity, time above intensity thresholds, and overall movement volume. Cluster-derived measures are better aligned with questions about when activity occurs, whether daily profiles follow recognizable temporal shapes, and whether particular patterns of activity across the day are associated with health outcomes. In this study, the cluster-based measures did not produce uniformly larger model fit improvements, but they performed comparably while preserving an interpretable representation of daily temporal structure. This comparable performance is reassuring for an alternative measure intended to address questions about within-day activity patterns, but it does not establish added clinical utility.

The sorting analyses further illustrate how preprocessing choices can be used to align the clustering approach with different scientific questions. Without sorting, clusters represent activity patterns tied to specific clock times, and the corresponding regression coefficients describe associations between outcomes and the proportion of days spent in those clock-time activity patterns. Sorting the entire day changes the interpretation by emphasizing sustained activity levels regardless of when they occurred, making the resulting measures closer to distributional or intensity-based summaries. Sorting within time segments provides an intermediate option: it relaxes exact ordering within a period while retaining some information about whether activity occurred in the morning, afternoon, or evening. Thus, sorting is not merely a technical sensitivity analysis; it defines the degree of temporal invariance permitted by the representation. Other pairwise similarity frameworks, including dynamic time warping or Gaussian-process–based approaches, could be combined with hierarchical clustering. Dynamic time warping defines similarity by allowing temporal features to shift to improve alignment and is valuable when overall profile shape is of primary interest and precise timing is treated as nuisance variation. In the present study, however, broad clock-time placement was part of the construct of interest, while only limited within-period timing variation was intentionally relaxed. The selected fixed-profile and within-segment sorting approaches therefore reflect the scientific objective rather than an arbitrary computational partition.

Because cluster-derived measures depend on analytic choices made before and during clustering, transparent reporting is important for interpretation and comparison across studies. Jones et al [20] emphasized that clustering applications require clear reporting of methodological choices, including the underlying machine learning method and tuning decisions. For accelerometer-derived clustering, useful reporting elements include the analytic window, preprocessing pipeline, nonwear and device-malfunction rules, exclusion criteria, missing-data handling, sorting or transformation strategy, clustering algorithm, distance or similarity measure, method for selecting the number of clusters, random seeds or initialization settings, regression model specifications, and code availability. These details do not guarantee that another sample will yield the same clusters, but they allow the analysis to be evaluated, repeated within the same dataset, and compared across studies. This distinction matters: reproducibility within the same dataset concerns whether the same pipeline can be rerun with the same data, whereas external replicability concerns whether similar patterns and associations are observed across populations, devices, monitoring durations, and study settings.

Several limitations provide context for these findings. First, the present analysis was conducted at baseline within the Stanford GOALS trial and was not designed to evaluate longitudinal changes, intervention effects, or causal relationships. Although GOALS is an intervention trial, the objective of this manuscript is methodological: to derive and evaluate cluster-based accelerometer summaries in the baseline data. Second, the analysis was based on 1 week of accelerometer monitoring per participant. Longer monitoring periods may reveal additional within-person variability and could affect the stability of cluster-derived summaries. Third, the analytic window was selected to retain broad daytime and evening coverage while reducing loss of usable profiles due to nonwear or device malfunction. This standardized representation was necessary for aligned daily-profile clustering, but it also required excluding profiles with nonwear or device malfunction within the analytic window. If missingness was related to activity behavior or cardiometabolic outcomes, this complete-case approach could affect estimated associations. These considerations support prespecification of analytic windows, exclusion rules, preprocessing decisions, and missing-data handling in future applications.

The clustering results may also be sample dependent. Patterns discovered in one dataset can reflect characteristics of the sample, device, monitoring protocol, preprocessing pipeline, and selected clustering method. The number of clusters is one especially consequential choice. We used prediction strength to guide selection of k, but other approaches, such as the Calinski-Harabasz index or silhouette index, could lead to different solutions [34,35]. In addition, the uncertainty associated with deriving clusters was not fully propagated into the downstream regression models. Resampling or bootstrap-based approaches may help evaluate the stability of discovered patterns and the uncertainty of their associations with outcomes. Generalizability is also bounded by the study population and design: participants were Latino children with overweight or obesity from low-income families, monitored over a 1-week period in a baseline cross-sectional setting. External validation is needed to determine how well these activity-pattern summaries transfer to other populations, devices, monitoring durations, and settings.

Future work can extend this framework in several directions. Within GOALS and similar studies, longitudinal and intervention analyses could evaluate whether baseline cluster-derived summaries predict change in cardiometabolic outcomes or whether changes in activity-pattern membership correspond to intervention response. External validation studies can assess whether similar temporal patterns emerge in other cohorts and with other accelerometer-processing pipelines. A systematic methodological comparison of fixed-alignment clustering with dynamic time warping or Gaussian-process–based similarity measures followed by hierarchical clustering could further characterize the tradeoff between preserving clock-time interpretability and allowing elastic shape-based alignment. Individual-level clustering may also be useful when the research question concerns deviations from a person’s usual activity pattern rather than differences across participants. Finally, the same general profile-based logic could be evaluated using other accelerometer scales, such as monitor-independent movement summary measures or raw acceleration signals, and possibly other intensively sampled behavioral or sensor-derived time series.

In conclusion, cluster-derived daily activity measures provide a regression-ready way to summarize accelerometer profiles while retaining information about both activity magnitude and within-day timing. In this baseline methodological analysis, these measures performed comparably to traditional accelerometer summaries while capturing a different aspect of physical activity behavior: the shape and timing of daily movement patterns. The approach is therefore best viewed as a complement to existing accelerometer metrics rather than as a demonstrably superior predictor of the cardiometabolic outcomes examined here. With transparent reporting, external validation across diverse populations and settings, and further evaluation in longitudinal and intervention analyses, cluster-derived profile measures may help expand how accelerometer data are used to study relationships between daily activity patterns and health outcomes.

## Supplementary material

10.2196/87532 Multimedia Appendix 1 Supplementary figures and tables describing accelerometer preprocessing, analytic-window selection, activity summary measures, and clustering diagnostics.

## Abbreviations

AIC: Akaike information criterion
AIM: Activity Intensity Mean
AIV: Activity Intensity Variability
CPM: counts per minute
LPA: light physical activity
MPA: moderate physical activity
MVPA: moderate-to-vigorous physical activity
PCA: principal component analysis
SB: sedentary behavior
TAM: Time Active Mean
TAV: Time Active Variability
VM: vector magnitude
VPA: vigorous physical activity

## References

[1] Troiano RP, McClain JJ, Brychta RJ, Chen KY (2014). Evolution of accelerometer methods for physical activity research. Br J Sports Med.

[2] Neishabouri A, Nguyen J, Samuelsson J (2022). Quantification of acceleration as activity counts in ActiGraph wearable. Sci Rep.

[3] Arigo D, Mogle JA, Brown MM (2020). Differences between accelerometer cut point methods among midlife women with cardiovascular risk markers. Menopause.

[4] Banda JA, Haydel KF, Davila T (2016). Effects of varying epoch lengths, wear time algorithms, and activity cut-points on estimates of child sedentary behavior and physical activity from accelerometer data. PLoS ONE.

[5] Brailey G, Metcalf B, Lear R, Price L, Cumming S, Stiles V (2022). A comparison of the associations between bone health and three different intensities of accelerometer-derived habitual physical activity in children and adolescents: a systematic review. Osteoporos Int.

[6] Bruijns BA, Truelove S, Johnson AM, Gilliland J, Tucker P (2020). Infants’ and toddlers’ physical activity and sedentary time as measured by accelerometry: a systematic review and meta-analysis. Int J Behav Nutr Phys Act.

[7] Leroux A, Di J, Smirnova E (2019). Organizing and analyzing the activity data in NHANES. Stat Biosci.

[8] Romanzini M, Petroski EL, Ohara D, Dourado ACR, Reichert FF (2014). Calibration of ActiGraph GT3X, Actical and RT3 accelerometers in adolescents. Eur J Sport Sci.

[9] Rowlands AV, Rennie K, Kozarski R (2014). Children’s physical activity assessed with wrist-and hip-worn accelerometers. Med Sci Sports Exerc.

[10] Santos-Lozano A, Marín PJ, Torres-Luque G, Ruiz JR, Lucía A, Garatachea N (2012). Technical variability of the GT3X accelerometer. Med Eng Phys.

[11] Zhang S, Rowlands AV, Murray P, Hurst TL (2012). Physical activity classification using the GENEA wrist-worn accelerometer. Med Sci Sports Exerc.

[12] Bammann K, Thomson NK, Albrecht BM, Buchan DS, Easton C (2021). Generation and validation of ActiGraph GT3X+ accelerometer cut-points for assessing physical activity intensity in older adults. The OUTDOOR ACTIVE validation study. PLOS ONE.

[13] Colley RC, Tremblay MS (2011). Moderate and vigorous physical activity intensity cut-points for the Actical accelerometer. J Sports Sci.

[14] Evenson KR, Herring AH, Wen F (2017). Accelerometry-assessed latent class patterns of physical activity and sedentary behavior with mortality. Am J Prev Med.

[15] Fraysse F, Post D, Eston R, Kasai D, Rowlands AV, Parfitt G (2021). Physical activity intensity cut-points for wrist-worn GENEActiv in older adults. Front Sports Act Living.

[16] Kim Y, Beets MW, Welk GJ (2012). Everything you wanted to know about selecting the “right” Actigraph accelerometer cut-points for youth, but…: a systematic review. J Sci Med Sport.

[17] Robinson TN, Matheson D, Desai M (2013). Family, community and clinic collaboration to treat overweight and obese children: Stanford GOALS-A randomized controlled trial of a three-year, multi-component, multi-level, multi-setting intervention. Contemp Clin Trials.

[18] Robinson TN, Matheson D, Wilson DM (2021). A community-based, multi-level, multi-setting, multi-component intervention to reduce weight gain among low socioeconomic status Latinx children with overweight or obesity: the Stanford GOALS randomised controlled trial. Lancet Diabetes Endocrinol.

[19] Bai J, He B, Shou H, Zipunnikov V, Glass TA, Crainiceanu CM (2014). Normalization and extraction of interpretable metrics from raw accelerometry data. Biostatistics.

[20] Jones PJ, Catt M, Davies MJ (2021). Feature selection for unsupervised machine learning of accelerometer data physical activity clusters—a systematic review. Gait Posture.

[21] Nawrin SS, Inada H, Momma H, Nagatomi R (2024). Examining physical activity clustering using machine learning revealed a diversity of 24-hour step-counting patterns. J Act Sedentary Sleep Behav.

[22] Aqeel M, Guo J, Lin L (2021). Temporal physical activity patterns are associated with obesity in U.S. adults. Prev Med.

[23] Nawrin SS, Inada H, Momma H, Nagatomi R (2024). Twenty-four-hour physical activity patterns associated with depressive symptoms: a cross-sectional study using big data-machine learning approach. BMC Public Health.

[24] Niemelä M, Kangas M, Farrahi V (2019). Intensity and temporal patterns of physical activity and cardiovascular disease risk in midlife. Prev Med.

[25] Smagula SF, Boudreau RM, Stone K (2015). Latent activity rhythm disturbance sub-groups and longitudinal change in depression symptoms among older men. Chronobiol Int.

[26] Smagula SF, Krafty RT, Thayer JF, Buysse DJ, Hall MH (2018). Rest-activity rhythm profiles associated with manic-hypomanic and depressive symptoms. J Psychiatr Res.

[27] Smagula SF, Zhang G, Gujral S (2022). Association of 24-hour activity pattern phenotypes with depression symptoms and cognitive performance in aging. JAMA Psychiatry.

[28] Choi L, Liu Z, Matthews CE, Buchowski MS (2011). Validation of accelerometer wear and nonwear time classification algorithm. Med Sci Sports Exerc.

[29] Padaco. GitHub.

[30] Tibshirani R, Walther G (2005). Cluster validation by prediction strength. J Comput Graph Stat.

[31] Yu J, Kapphahn K, Moore H, Haydel F, Robinson T, Desai M (2023). Prediction strength for clustering activity patterns using accelerometer data. J Meas Phys Behav.

[32] Expert Panel on Integrated Guidelines for Cardiovascular Health and Risk Reduction in Children and Adolescents, National Heart, Lung, and Blood Institute (2011). Expert panel on integrated guidelines for cardiovascular health and risk reduction in children and adolescents: summary report. Pediatrics.

[33] Kwac J, Flora J, Rajagopal R (2014). Household energy consumption segmentation using hourly data. IEEE Trans Smart Grid.

[34] Caliński T, Harabasz J (1974). A dendrite method for cluster analysis. Commun Stat.

[35] Rousseeuw PJ (1987). Silhouettes: a graphical aid to the interpretation and validation of cluster analysis. J Comput Appl Math.

