# Supplementary material for: Clustering-Based Accelerometer Measures of Physical Activity Patterns in Children With Overweight or Obesity: Baseline Cross-Sectional Methodological Analysis
Source: JMIR Form Res. 2026 Oct 8;10:e87532. doi: 10.2196/87532 (PMC13649174; doi:10.2196/87532)
Supplement: Supplementary file 1 [file formative-v10-e87532-s001.docx]

**SUPPLEMENTARY MATERIAL**

**Figure S1.** Distribution of within-participant standard deviations used for data-quality screening, following the general approach described by Bai et al. (2014). The upper panel shows the distribution of standard deviations across participants’ daily profiles, and the lower panel shows the corresponding distribution after applying the candidate threshold of c=0.6. The vertical reference lines indicate c=0.6.

**Figure S2.** **PCA projections of clustered 96-bin daily activity profiles across preprocessing specifications.** Each point represents one valid daily activity profile projected onto the first two principal components, with color indicating assigned k-means cluster. Panels show the unsorted profiles and sorted preprocessing approaches using one full-day interval (Sort_01), two 8-hour intervals (Sort_02), four 4-hour intervals (Sort_04), eight 2-hour intervals (Sort_08), and sixteen 1-hour intervals (Sort_16). PCA was performed separately for each preprocessing specification and was used only as a post hoc visual diagnostic. The projections generally show continuous activity-profile variation, with assigned clusters occupying different regions of the reduced two-dimensional space.

**Unsorted**

**Sort_01**

**Sort_02**

**Sort_04**

**Sort_08**

**Sort_16**

**Table S1**: Candidate time frames used for data-quality screening and analytic-window selection. Choi’s method was used to identify non-wear at 1-minute intervals in the count data (Choi, 2011). Additional device malfunctions were identified by iterative clustering of raw acceleration signals and review of outlying profiles. Confirmed malfunctions included activity spikes outside the normal range and prolonged constant non-zero values consistent with sensor sticking. For each candidate time window, the table reports the number of participants with 0, 1 or more, 2 or more, and up to 7 or more valid daily profiles after excluding profiles with non-wear or device malfunction within that window. This summary was used to select the analytic window that balanced broad daytime/evening coverage with participant-level profile availability.

| Candidate  time window | Participants with valid daily profiles after exclusion, n* | | | | | | | |
| --- | --- | --- | --- | --- | --- | --- | --- | --- |
|  | 0 | 1+ | 2+ | 3+ | 4+ | 5+ | 6+ | 7+ |
| 05:00-17:00 | 7 | 261 | 257 | 255 | 248 | 228 | 212 | 145 |
| 05:00-18:00 | 7 | 261 | 257 | 255 | 243 | 225 | 211 | 138 |
| 05:00-19:00 | 7 | 261 | 257 | 253 | 239 | 222 | 205 | 129 |
| 05:00-20:00 | 7 | 261 | 255 | 249 | 238 | 220 | 198 | 114 |
| 05:00-21:00 | 9 | 259 | 254 | 247 | 232 | 216 | 187 | 88 |
| 05:00-22:00 | 9 | 259 | 251 | 241 | 228 | 203 | 158 | 62 |
| 05:00-23:00 | 12 | 256 | 245 | 234 | 218 | 183 | 122 | 39 |
| 05:00-24:00 | 12 | 256 | 245 | 234 | 218 | 183 | 122 | 39 |
| 06:00-18:00 | 7 | 261 | 257 | 255 | 243 | 225 | 211 | 138 |
| 06:00-19:00 | 7 | 261 | 257 | 253 | 239 | 222 | 205 | 129 |
| 06:00-20:00 | 7 | 261 | 255 | 249 | 238 | 220 | 198 | 114 |
| 06:00-21:00 | 9 | 259 | 254 | 247 | 232 | 216 | 187 | 88 |
| 06:00-22:00 | 9 | 259 | 251 | 241 | 228 | 203 | 158 | 62 |
| 06:00-23:00 | 12 | 256 | 245 | 234 | 218 | 183 | 122 | 39 |
| 06:00-24:00 | 12 | 256 | 245 | 234 | 218 | 183 | 122 | 39 |
| 07:00-19:00 | 7 | 261 | 257 | 253 | 239 | 222 | 206 | 129 |
| 07:00-20:00 | 7 | 261 | 255 | 249 | 238 | 220 | 199 | 114 |
| 07:00-21:00 | 9 | 259 | 254 | 247 | 232 | 216 | 188 | 88 |
| 07:00-22:00 | 9 | 259 | 251 | 241 | 228 | 203 | 159 | 62 |
| 07:00-23:00 | 12 | 256 | 245 | 234 | 218 | 183 | 123 | 39 |
| 07:00-24:00 | 12 | 256 | 245 | 234 | 218 | 183 | 123 | 39 |
| 08:00-20:00 | 5 | 263 | 261 | 255 | 247 | 229 | 207 | 118 |
| 08:00-21:00 | 5 | 263 | 259 | 251 | 244 | 221 | 192 | 93 |
| 08:00-22:00 | 6 | 262 | 256 | 247 | 235 | 207 | 164 | 64 |
| 08:00-23:00 | 7 | 261 | 249 | 238 | 224 | 186 | 128 | 39 |
| 08:00-24:00 | 7 | 261 | 249 | 238 | 224 | 186 | 128 | 39 |
| 09:00-21:00 | 3 | 265 | 260 | 255 | 247 | 223 | 195 | 94 |
| 09:00-22:00 | 4 | 264 | 257 | 252 | 237 | 209 | 165 | 64 |
| 09:00-23:00 | 7 | 261 | 252 | 241 | 227 | 187 | 128 | 40 |
| 09:00-24:00 | 7 | 261 | 252 | 241 | 227 | 187 | 128 | 40 |
| 10:00-22:00 | 3 | 265 | 257 | 253 | 238 | 210 | 167 | 65 |
| 10:00-23:00 | 6 | 262 | 252 | 241 | 228 | 189 | 129 | 41 |
| 10:00-24:00 | 6 | 262 | 252 | 241 | 228 | 189 | 129 | 41 |
| 11:00-23:00 | 6 | 262 | 253 | 242 | 229 | 192 | 130 | 43 |
| 11:00-24:00 | 6 | 262 | 253 | 242 | 229 | 192 | 130 | 43 |
| 12:00-24:00 | 4 | 264 | 256 | 242 | 229 | 193 | 131 | 45 |

*Columns 1+ through 7+ indicate the number of participants with at least the specified number of valid daily profiles after excluding profiles with non-wear or device malfunction within the candidate time window.

**Table S2:** Health outcomes and daily activity summary measures of a selection of Stanford GOALS participants.

| Participant | Age (years) | Is Male? | Day of Week | Day | Waist (cm) | Triglyceride (mg/dL) | Insulin (µIU/mL) | TAM | TAV | AIM | AIV | SB (min) | LPA (min) | MPA (min) | VPA (min) | MVPA (min) |
| --- | --- | --- | --- | --- | --- | --- | --- | --- | --- | --- | --- | --- | --- | --- | --- | --- |
| 1 | 10.46 | 0 | 0 | Sun | 76.40 | 95 | 9.6 | 0.00270 | 0.05193 | 0.00042 | 0.01458 | 567 | 315 | 66 | 12 | 78 |
| 1 | 10.46 | 0 | 1 | Mon | 76.40 | 95 | 9.6 | 0.00220 | 0.04689 | 0.00022 | 0.00959 | 694 | 202 | 50 | 14 | 64 |
| 1 | 10.46 | 0 | 3 | Wed | 76.40 | 95 | 9.6 | 0.00100 | 0.03161 | 0.00003 | 0.00281 | 698 | 213 | 45 | 3 | 48 |
| 1 | 10.46 | 0 | 5 | Fri | 76.40 | 95 | 9.6 | 0.00361 | 0.05998 | 0.00031 | 0.01046 | 608 | 267 | 59 | 26 | 85 |
| 1 | 10.46 | 0 | 6 | Sat | 76.40 | 95 | 9.6 | 0.00093 | 0.03042 | 0.00006 | 0.00436 | 747 | 188 | 21 | 4 | 25 |
| 2 | 10.16 | 1 | 0 | Sun | 102.00 | 124 | 25.4 | 0.00865 | 0.09259 | 0.00088 | 0.01731 | 467 | 300 | 114 | 79 | 193 |
| 2 | 10.16 | 1 | 1 | Mon | 102.00 | 124 | 25.4 | 0.00291 | 0.05384 | 0.00019 | 0.00822 | 525 | 305 | 95 | 35 | 130 |
| 2 | 10.16 | 1 | 2 | Tue | 102.00 | 124 | 25.4 | 0.00406 | 0.06356 | 0.00030 | 0.01088 | 540 | 285 | 93 | 41 | 134 |
| 2 | 10.16 | 1 | 3 | Wed | 102.00 | 124 | 25.4 | 0.00291 | 0.05384 | 0.00025 | 0.01151 | 624 | 222 | 78 | 36 | 114 |
| 2 | 10.16 | 1 | 5 | Fri | 102.00 | 124 | 25.4 | 0.00931 | 0.09606 | 0.00046 | 0.01111 | 478 | 304 | 107 | 71 | 178 |
| 2 | 10.16 | 1 | 6 | Sat | 102.00 | 124 | 25.4 | 0.01320 | 0.11415 | 0.00221 | 0.03207 | 425 | 290 | 118 | 127 | 245 |
| 3 | 11.03 | 0 | 0 | Sun | 119.30 | 73 | 28.2 | 0.00217 | 0.04650 | 0.00025 | 0.01011 | 506 | 363 | 73 | 17 | 90 |
| 3 | 11.03 | 0 | 1 | Mon | 119.30 | 73 | 28.2 | 0.00200 | 0.04468 | 0.00005 | 0.00258 | 590 | 295 | 37 | 38 | 75 |
| 3 | 11.03 | 0 | 3 | Wed | 119.30 | 73 | 28.2 | 0.00120 | 0.03467 | 0.00011 | 0.00536 | 526 | 349 | 66 | 17 | 83 |
| 3 | 11.03 | 0 | 4 | Thur | 119.30 | 73 | 28.2 | 0.00104 | 0.03219 | 0.00008 | 0.00504 | 622 | 263 | 56 | 18 | 74 |
| 3 | 11.03 | 0 | 5 | Fri | 119.30 | 73 | 28.2 | 0.00354 | 0.05937 | 0.00038 | 0.01098 | 561 | 277 | 92 | 30 | 122 |
| 3 | 11.03 | 0 | 6 | Sat | 119.30 | 73 | 28.2 | 0.00078 | 0.02788 | 0.00000 | 0.00028 | 554 | 334 | 65 | 7 | 72 |
| : | : | : | : |  | : | : | : | : | : | : | : | : | : | : | : | : |

**Table S3:** Health outcomes and participant-level averages of daily activity summary measures of a selection of Stanford GOALS participants.

| Participant | Age (years) | Is Male? | Waist (cm) | Triglyceride (mg/dL) | Insulin (µIU/mL) | Mean TAM | Mean TAV | Mean AIM | Mean AIV | Mean SB (min) | Mean LPA (min) | Mean MPA (min) | Mean VPA (min) | Mean MVPA (min) |
| --- | --- | --- | --- | --- | --- | --- | --- | --- | --- | --- | --- | --- | --- | --- |
| 1 | 10.46 | 0 | 76.40 | 95 | 9.6 | 0.00209 | 0.04417 | 0.00021 | 0.00836 | 662.80 | 237.00 | 48.20 | 11.80 | 60.00 |
| 2 | 10.16 | 1 | 102.00 | 124 | 25.4 | 0.00684 | 0.07901 | 0.00072 | 0.01518 | 509.83 | 284.33 | 100.83 | 64.83 | 165.67 |
| 3 | 11.03 | 0 | 119.30 | 73 | 28.2 | 0.00179 | 0.04088 | 0.00015 | 0.00573 | 559.83 | 313.50 | 64.83 | 21.17 | 86.00 |
| : | : | : | : | : | : | : | : | : | : | : | : | : | : | : |

**Table S4**: Pearson correlation matrix of summary measures.

|  | **Waist** | **Triglyceride** | **Insulin** | **TAM** | **TAV** | **AIM** | **AIV** | **SB** | **LPA** | **MPA** | **VPA** | **MVPA** |
| --- | --- | --- | --- | --- | --- | --- | --- | --- | --- | --- | --- | --- |
| **Waist** | 1.0000 | 0.1965 | 0.4851 | -0.3362 | -0.3353 | -0.2525 | -0.2182 | 0.3839 | -0.3361 | -0.2407 | -0.2617 | -0.2758 |
| **Triglyceride** | 0.1965 | 1.0000 | 0.3403 | -0.1561 | -0.0976 | -0.1778 | -0.1466 | 0.0468 | 0.0056 | -0.0373 | -0.1192 | -0.0917 |
| **Insulin** | 0.4851 | 0.3403 | 1.0000 | -0.2859 | -0.2628 | -0.2397 | -0.1784 | 0.1878 | -0.0604 | -0.1821 | -0.2908 | -0.2665 |
| **TAM** | -0.3362 | -0.1561 | -0.2859 | 1.0000 | 0.9622 | 0.9313 | 0.7846 | -0.5645 | 0.2043 | 0.5517 | 0.8252 | 0.7727 |
| **TAV** | -0.3353 | -0.0976 | -0.2628 | 0.9622 | 1.0000 | 0.8869 | 0.8019 | -0.6076 | 0.2798 | 0.5916 | 0.7685 | 0.7560 |
| **AIM** | -0.2525 | -0.1778 | -0.2397 | 0.9313 | 0.8869 | 1.0000 | 0.9139 | -0.4802 | 0.1292 | 0.4759 | 0.7869 | 0.7133 |
| **AIV** | -0.2182 | -0.1466 | -0.1784 | 0.7846 | 0.8019 | 0.9139 | 1.0000 | -0.4696 | 0.1788 | 0.4586 | 0.6680 | 0.6311 |
| **SB** | 0.3839 | 0.0468 | 0.1878 | -0.5645 | -0.6076 | -0.4802 | -0.4696 | 1.0000 | -0.8519 | -0.8362 | -0.5739 | -0.7494 |
| **LPA** | -0.3361 | 0.0056 | -0.0604 | 0.2043 | 0.2798 | 0.1292 | 0.1788 | -0.8519 | 1.0000 | 0.5064 | 0.0871 | 0.2916 |
| **MPA** | -0.2407 | -0.0373 | -0.1821 | 0.5517 | 0.5916 | 0.4759 | 0.4586 | -0.8362 | 0.5064 | 1.0000 | 0.6717 | 0.8871 |
| **VPA** | -0.2617 | -0.1192 | -0.2908 | 0.8252 | 0.7685 | 0.7869 | 0.6680 | -0.5739 | 0.0871 | 0.6717 | 1.0000 | 0.9378 |
| **MVPA** | -0.2758 | -0.0917 | -0.2665 | 0.7727 | 0.7560 | 0.7133 | 0.6311 | -0.7494 | 0.2916 | 0.8871 | 0.9378 | 1.0000 |

The Pearson correlations indicated the following patterns:

1. Waist and Insulin were moderately positively correlated (0.4851).
2. Waist circumference was weakly positively correlated with Triglycerides levels (0.1966).
3. TAM, TAV, AIM, and AIV were positively correlated with each other, with TAM and TAV having the strongest correlation (0.9622).
4. SB (sedentary behavior) has a strong negative correlation with LPA, MPA, VPA, and MVPA; most notably with LPA (-0.8519) and MVPA (-0.7494). Time spent sedentary directly reduces the time available to spend in the other activity categories, i.e. time spent in light, moderate, vigorous, and combined moderate-to-vigorous physical activity tends to decrease with increased SB.
5. MPA, VPA, and MVPA are positively correlated with each other, with the strongest correlation between VPA and MVPA (0.9378). These categories tend to increase or decrease together.
